# Diagnostic and Therapeutic Approach in Pediatric Pulmonary Abscess: Two Cases and Literature Review

**DOI:** 10.3390/jcm13247790

**Published:** 2024-12-20

**Authors:** Mariana Costin, Eliza Cinteză, Veronica Marcu, Mirela Luminița Pavelescu, Paraschiva Cherecheș-Panța, Julia Susanne Bălănescu, Ramona Elena Slăvulete, Taraș Roxana, Marcela Daniela Ionescu

**Affiliations:** 1 Department of Pediatrics, Carol Davila University of Medicine and Pharmacy, 020021 Bucharest, Romania; mariana.costin@umfcd.ro (M.C.); eliza.cinteza@umfcd.ro (E.C.); mirela.pavelescu@umfcd.ro (M.L.P.); daniela.ionescu@umfcd.ro (M.D.I.); 2 Department of Pediatric Nephrology, “M.S. Curie” Emergency Clinical Hospital for Children, 077120 Bucharest, Romania; 3 Department of Pediatric Cardiology, “Marie Curie” Emergency Children’s Hospital, 041451 Bucharest, Romania; 4 Department of Radiology, “M.S. Curie” Children’s Hospital, Constantin Brâncoveanu Boulevard, No. 20, 4th District, 041451 Bucharest, Romania; v.m.marcu@gmail.com; 5 Department of Pediatrics, “Grigore Alexandrescu” Emergency Children’s Hospital, 011743 Bucharest, Romania; 6 Department of Pediatrics, “Iuliu Hatieganu” University of Medicine and Pharmacy, 400124 Cluj-Napoca, Romania; pusacherechespanta@gmail.com; 7 Clinical Hospital for Pediatric Emergencies, 400124 Cluj-Napoca, Romania; 8 Emergency Clinical Hospital for Children “M.S. Curie”, 041451 Bucharest, Romania; julia-susanne.balanescu@rez.umfcd.ro; 9 National Institute for Infectious Diseases “Prof. Dr. Matei Balș”, 021105 Bucharest, Romania; ramona-elena.slavulete@rez.umfcd.ro

**Keywords:** pulmonary abscess, children, antibiotics, video-assisted thoracic surgery (VATS), thoracoscopy, empyema

## Abstract

Pulmonary abscess is a rare but serious condition in pediatric patients, caused by severe pulmonary infection that leads to tissue destruction and necrosis. It can be classified as primary or secondary depending on the cause. Establishing an etiology in pediatric pulmonary abscesses is challenging, underscoring the essential role of advanced imaging techniques, such as computed tomography, in achieving an accurate diagnosis and differentiating among various conditions that may mimic lung abscess. While conservative management with antibiotics is the first line of treatment, some cases may progress and require surgical intervention. We present two clinical cases of pediatric lung abscesses, emphasizing the importance of timely intervention, accompanied by a brief review of current knowledge that highlights key clinical features, diagnostic challenges, and therapeutic approaches in pediatric lung abscess.

## 1. Introduction

### 1.1. Definition

Childhood lung abscess is a relatively rare condition, which contributes to the scarcity of quality data around it [1].

Lung abscess is a possible local complication of community-acquired pneumonia and, while it can lead to severe illness, extended hospital stays, and a prolonged disease course, the majority of patients typically achieve full recovery [2].

A lung abscess arises from a pulmonary infection causing extensive destruction of lung parenchyma, resulting in the development of a circumscribed, thick-walled cavity containing purulent material due to tissue suppuration and necrosis [3].

### 1.2. Classification 

Lung abscesses can be classified by several factors, including etiology, duration, or the pathophysiologic process by which they spread from extrapulmonary sites. Acute abscesses are present for less than 4–6 weeks, while chronic abscesses persist for longer periods of time [4].

Regarding etiology, lung abscesses can be classified as either primary or secondary, depending on the presence of predisposing factors. Primary abscess appears in previously healthy children, whereas secondary abscess can occur due to underlying local or systemic causes. Secondary lung abscess develop in patients with inherited lung diseases, such as congenital pulmonary airway malformation, cystic fibrosis, or primary ciliary dyskinesia, as well as in patients with immunocompromised conditions like immunodeficiencies [1,4]. It can develop in children with previously acquired disorders such as inhaled foreign body or pulmonary metastasis [5,6]. 

Additionally, lung abscesses can result from extrapulmonary sites through hematogenous spread, as in septic thromboembolisms or by direct extension from abdominal or oropharyngeal sites [4,7].

In children with neurodevelopmental delay, pulmonary aspiration plays a key role in the formation of lung abscesses due to impaired clearance and poorly coordinated swallowing. It is important to identify these predisposing factors as certain conditions are more frequently associated with specific microorganisms [1,7].

### 1.3. Etiology and Pathogenesis

Anaerobic bacteria were consistently identified in the past as the primary pathogens in lung abscesses, Peptostreptococcus species, Bacteroides species, and Fusobacterium species being among the most common [2]. Recent studies suggest a shift in bacteriological characteristics, with Staphylococcus aureus and Streptococcus species being the most common, especially in primary abscesses, followed by some Gram-negative bacilli such as Klebsiella pneumoniae [8,9,10]. Secondary abscesses are more frequently caused by anaerobes and Gram-positive cocci [2,7]. Nonbacterial and atypical bacterial pathogens, such as parasites, fungi, and Mycobacterium species, can lead to lung abscesses, usually in immunocompromised individuals [11].

Pulmonary abscesses commonly develop in the right lung, particularly in the posterior segment of the right upper lobe or the superior region of the right lower lobe, due to gravitational flow of infectious material and the vertical orientation of the right bronchus [4].

The infectious material settling in the distal bronchi initiates local inflammation in the lung tissue. Over a span of hours to a few days, this inflammation escalates due to the accumulation of exudate, blood cells, and necrotic tissue, which may progress to liquefactive necrosis, culminating in abscess formation. As necrotic material is expelled through the bronchus, a necrotic cavity containing an air–fluid level develops. The infection can potentially spread into the pleural space, leading to empyema without necessarily rupturing the abscess cavity [4].

### 1.4. Clinical Features

The clinical presentation of pulmonary abscess in children can vary widely depending on the age of the child, the underlying etiology, and the extent of lung involvement. Children may exhibit symptoms for several days [4,12]. Typically, they present with a high fever and cough, which can be productive or with purulent sputum, and often have associated loss of appetite, are ill appearing, and caregivers may report weight loss. Rarely, chest pain, dyspnea, and hemoptysis are present. In newborns, infants, immunocompromised patients, or patients undergoing long-term cortisone treatment, fever may be absent [4].

Children with a pulmonary abscess may present with nonspecific symptoms, especially newborns and infants, such as apnea, malaise, vomiting, diarrhea, and hematemesis [4].

Clinical examination can reveal dullness on percussion and diminished or absent breath sounds in the affected area. Fine or coarse crackles may be heard, particularly over areas of consolidation or cavitation; cavernous or amphoric murmurs have been described in the literature as suggestive clinical signs however they are very rarely identified in practice. Dullness on percussion and absence of breath sounds are noted in lung abscesses when associated with or complicated by massive pleural effusions. In rare cases of centrally located abscesses or in the presence of small abscesses, the clinical examination of the thorax may be normal [2,13].

In severe cases, respiratory distress may develop, manifesting as tachypnea, nasal flaring, intercostal or subcostal retractions, and cyanosis. This indicates significant compromise of lung function and requires urgent medical attention [14,15].

Depending on the underlying cause and extent of infection, children with pulmonary abscess may present with extra-pulmonary manifestations. These can include symptoms and signs of predisposing conditions such as neurological impairment or congenital anomalies that increase the risk of aspiration. Additionally, signs of systemic complications including the dissemination of infection may also be observed [16].

The clinical presentation of lung abscesses in children can resemble other respiratory conditions such as pneumonia and bronchiolitis, while less common disorders also require consideration for a proper differential diagnosis. Therefore, a thorough evaluation, including detailed history, physical examination, and appropriate diagnostic tests such as imaging and microbiological studies, is essential for accurate diagnosis and effective management [5,6,17].

### 1.5. Diagnostic Evaluation

Imaging is the cornerstone of diagnosing pulmonary abscesses; however, additional tests such as a complete blood count, inflammatory markers, and bacterial cultures are essential for a comprehensive diagnostic evaluation. Laboratory tests typically reveal leukocytosis with neutrophilia, elevated acute phase reactants (such as C-reactive protein, fibrinogen, and procalcitonin), a low hemoglobin level, hypoalbuminemia, and elevated D-dimer levels, all indicative of the systemic inflammatory response seen in severe pulmonary infections. Blood cultures can establish the etiology and should be performed even in afebrile newborns, young infants, and children with deficient immune status, although the yield of positive results is poor [18].

Sputum culture and sensitivity testing may be used to determine the causative organism. However, it is rarely reliable due to the high risk of contamination with bacteria from upper airway flora. Bronchoalveolar lavage (BAL) or lung biopsy may be necessary to identify the causative organism and guide appropriate antimicrobial therapy, particularly in cases of treatment failure or recurrent abscesses [16].

Standard microbiological methods fail to identify the etiological cause in nearly half of pulmonary abscess cases [9,19].

However, in lung abscess, the diagnosis is based on imaging studies. Chest radiography is usually the initial imaging modality of choice, revealing characteristic findings such as circumscribed opacity often containing an air–fluid level. Consolidation and an associated pleural effusion can be identified, but chest radiography underestimates the degree of parenchymal destruction. Radiographs must be taken with the patients in a decubitus or upright position because a supine film may not show an air–fluid level [20,21].

Chest ultrasound is effective in distinguishing lung abscesses from pleural collections or parenchymal abnormalities and can be useful for follow-up. The procedure is painless, radiation free, and can be performed without sedation. However, its accuracy may be limited by intervening aerated lung tissue, and its success depends on the operator’s skill [22].

Computed tomography (CT) provides detailed anatomical information that surpasses chest radiography, is crucial for identifying complications, and may provide further details in cases of atypical presentations. Although a lung abscess is identifiable on chest radiography, CT, as the most sensitive and specific imaging modality, can differentiate between parenchymal lesions and pleural collections, each of which requires a distinct management approach [22]. It also assists in identifying the underlying cause of the abscess, whether it is an infected cyst, congenital anomaly, airway obstruction, or adjacent structure infection. Additionally, CT plays a key role in surgical planning by guiding decision-making for resection [12].

Moreover, chest CT and ultrasound are valuable tools for guiding interventional procedures, such as chest tube placement and needle aspiration for diagnosis and drainage, as well as for surgical planning [21].

### 1.6. Management

Lung abscesses can be managed either conservatively with antibiotics and supportive care or surgically, depending on factors such as the abscess’s size, cause, and response to initial treatment [10].

### 1.7. Conservative Management

Extended antibiotic therapy demonstrates effectiveness in treating lung abscess in as many as 90% of pediatric cases, and the treatment outcome for primary lung abscess is generally favorable. Morbidity and mortality usually result from conditions predisposing to secondary abscesses [7].

Intravenous antibiotic therapy typically lasts 2–3 weeks but may be extended based on disease progression. Usually, parenteral treatment is followed by oral therapy, totaling approximately 3–6 weeks of treatment [23]. The duration of antibiotic therapy varies according to local resistance patterns and experience. The choice of antimicrobial agents depends on factors such as the type of abscess and whether the child has any underlying conditions, the ability to isolate and identify the organism, costs, and local practices [2,7].

Current antibiotic regimens for lung abscess focuses on broad-spectrum antibiotic therapy targeting Gram-positive, Gram-negative, and anaerobic organisms [1]. Most cases can be effectively managed with antibiotics alone, with therapy typically starting with two antibiotic agents. Usually, clinical improvement appears in approximately one week, with fever typically improving after 3–4 days of antibiotic therapy, and the general condition improving after 4–7 days. From an imaging perspective, complete resolution of the radiographic image can be seen after 2–3 months. If a patient fails to respond to antibiotic treatment within 7–10 days, reassessment for resistant organisms or airway obstruction is necessary [4,24].

Antibiotics should target common organisms such as Staphylococcus aureus, Streptococcus pneumoniae, and Gram-negative bacilli typically present in the upper respiratory tract, particularly when treating primary abscess. In cases where there is a risk of secondary abscess formation due to aspiration, it is crucial to include antibiotics effective against anaerobes commonly found in the upper airway. In immunocompromised individuals, antibiotic coverage needs to be broader and may involve considering fungal pathogens [1,25].

Penicillin, traditionally the first choice antibiotic, is effective against streptococcal species, but nowadays resistance can be an issue due to beta-lactamase-producing organisms. Acceptable treatment options for the majority of patients typically involve either a beta-lactam–beta-lactamase inhibitor combination or a carbapenem. Third-generation cephalosporins are preferred to cover Gram-positive and Gram-negative microbes, in addition to an antibiotic such as Clindamycin, which offers the advantage of covering Staphylococcus aureus and anaerobes [10]. In areas with high rates of methicillin-resistant S. aureus, Vancomycin is recommended until culture results are available [1,2].

The initial treatment plan may be modified as necessary, considering culture results and the patient’s response to therapy. It is important to note that anaerobic organisms are difficult to grow in culture, and susceptibility testing is therefore seldom conducted [2].

### 1.8. Surgical Treatment

If there is no evident clinical improvement after a week of appropriate antimicrobial treatment, additional imaging tests, preferably a CT scan, need to be performed to characterize possible complications such as empyema or new abscesses, and in case surgical intervention is anticipated [26].

Approximately 10% of individuals do not respond to antimicrobial treatment and require either a drainage procedure or surgical intervention [7]. Surgical options include percutaneous aspiration and chest tube placement, bronchoscopic drainage, video-assisted thoracoscopic surgery (VATS), or open surgery [12]. VATS is becoming increasingly favored as a therapeutic option because of better rates of success when compared to aspiration and drainage, while having lower rates of complications and faster rates of recovery than open surgery [23,27]. Procedures such as needle aspiration or percutaneous catheter drainage aid in both the diagnosis and serve as a therapeutic approach [28].

Transthoracic catheter drainage, conducted percutaneously, can be guided by imaging [29,30]. Depending on the resources, this may involve computed tomography, ultrasound, or fluoroscopy [12,31]. However, there is a slight chance of pleural space infection with these procedures, along with minor risks of hemorrhage or pneumothorax [32]. 

In cases where patients fail to respond to antibiotic therapy, with or without catheter drainage, or develop complications like significant hemorrhage or bronchopleural fistula, surgery may be necessary [23]. Contributing factors include large abscess size and antibiotic-resistant organisms. Video-assisted thoracic surgery (VATS) is preferred for its precision in removing infected material and reducing the risk of reintervention and the length of hospital stay [26,32]. If a bronchopleural fistula or extensive pulmonary necrosis is suspected, open surgery is likely required. The surgical resection is determined by the extent of the lesion, with lobectomy being the most common procedure performed [12].

### 1.9. Evolution, Complication, and Prognosis

The evolution of uncomplicated abscesses is usually favorable. Complications of lung abscesses in children can arise due to the spread of infection, persistent inflammation, and associated structural damage to the lung tissue [18].

Lung abscesses can lead to the formation of empyema, characterized by the accumulation of infected fluid within the pleural space. This can result in worsening respiratory distress, pleuritic chest pain, and systemic signs of sepsis [32].

Chronic or large abscesses may erode into adjacent bronchi and the pleural space, leading to the formation of a bronchopleural fistula. This communication between the airway and the pleural cavity can result in persistent air leakage, recurrent pneumothorax, and an increased risk of secondary infections [18].

Inflammatory processes associated with lung abscesses can lead to the development of pleural effusion, further compromising lung function and increasing the risk of respiratory compromise and respiratory failure [33].

Severe or untreated lung abscesses can result in bacteremia and systemic spread of infection, leading to sepsis, septic shock, and multi-organ dysfunction syndrome. This represents a life-threatening complication requiring urgent medical intervention [9].

Prolonged inflammation and infection within the lung parenchyma can lead to tissue necrosis and cavitation, increasing the risk of lung abscess enlargement, persistent symptoms, and impaired lung function [34].

Children with predisposing factors for pulmonary abscesses, such as impaired swallowing reflexes or neurological disorders, are at an increased risk of aspiration pneumonitis, which can progress to the development of lung abscesses if not promptly treated [1,35].

Recurrent or chronic lung abscesses can result in chronic lung disease, including bronchiectasis, fibrosis, and impaired lung function, predisposing children to recurrent respiratory infections and long-term respiratory complications [18].

Rare but serious complications of lung abscesses in children include the systemic embolization of septic emboli, resulting in metastatic infections in distant organs such as the brain, bones, or joints [36].

The long-term outcome of lung abscesses in children is generally favorable and most children recover uneventfully with no pulmonary sequelae, but the existence of underlying conditions will influence the prognosis [1]. Early recognition and prompt initiation of appropriate therapy are crucial to prevent complications and improve outcomes in affected children [37]. Multidisciplinary management involving pediatricians, pulmonologists, radiologists, pathologists, infectious disease specialists, and pediatric surgeons is often required to optimize outcomes and reduce the risk of complications [33].

#### 1.9.1. Case 1

A 2-year-old boy was admitted to the hospital presenting with fever, productive cough, and abdominal pain. The patient developed a cough one month prior to admission, which became productive and more frequent five days before admission, coinciding with the onset of fever. His perinatal history was unremarkable, although he had been hospitalized previously for pneumonia in the right lobe eight months earlier.

Upon admission, he was febrile with associated rigors, and although underweight, he had a normal stature according to the WHO percentiles. Mucocutaneous pallor was noted during the examination. Respiratory assessment revealed a frequent productive cough, dullness on percussion, diminished breath sounds, and fine crackles in the right lung, with a respiratory rate of 40 breaths per minute and normal oxygen saturation.

Blood analysis showed leukocytosis (19,600/µL) with neutrophilia (9900/µL) and anemia due to iron deficiency, exacerbated in the context of infection (hemoglobin 9.5 g/dL, serum iron 10.7 µg/dL) (Table 1). There were marked indications of an inflammatory syndrome with elevated CRP (120.4 mg/L) and procalcitonin (5.9 ng/mL), and arterial blood gas analysis showed no signs of hypoxemia. Stool and blood cultures were negative. To rule out Mycobacterium tuberculosis infection, an Interferon Gamma Release Assay (IGRA) and Tuberculin Skin Test were performed, both yielding negative results.

**Figure 1:**
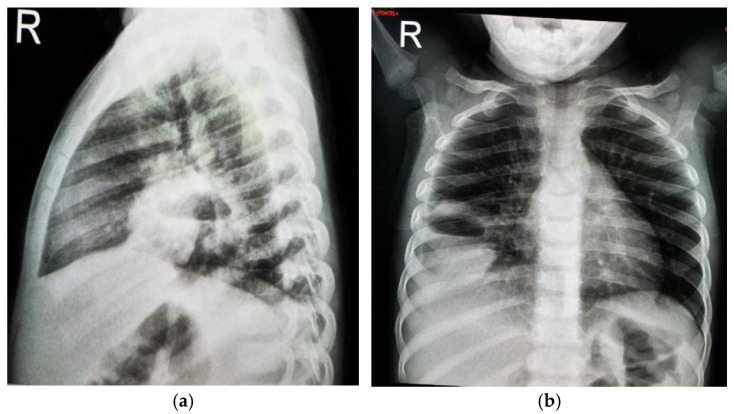
Two-year old male child with pulmonary abscess: oval mass with air–fluid level and blurred margins in right lower lobe; (**a**) lateral chest X-ray; (**b**) posteroanterior chest X-ray.

A chest X-ray revealed an oval mass with an air–fluid level and blurred margins in the right lower lobe, suggestive of a pulmonary abscess, and blunting of the costophrenic angle (Figure 1).

**Figure 2:**
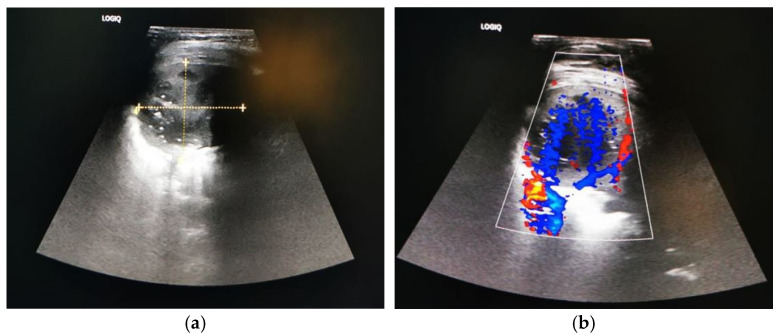
Two-year old male child with pulmonary abscess: thoracic ultrasonography showing (**a**) round–oval lesion with heterogeneous anechoic content, with no adjacent pleural effusion; (**b**) absent intrinsic vascular signal, but peripheral parietal vascular tract is present, suggestive of underlying congenital pulmonary airway malformation.

Thoracic ultrasonography indicated a minimal pleural reaction, and the presence of a peripheral parietal vascular tract led to the suspicion of an underlying congenital pulmonary airway malformation (Figure 2). Broad-spectrum antibiotic therapy with Meropenem (120 mg/kg/day) and Clindamycin (30 mg/kg/day) was initiated.

**Figure 3:**
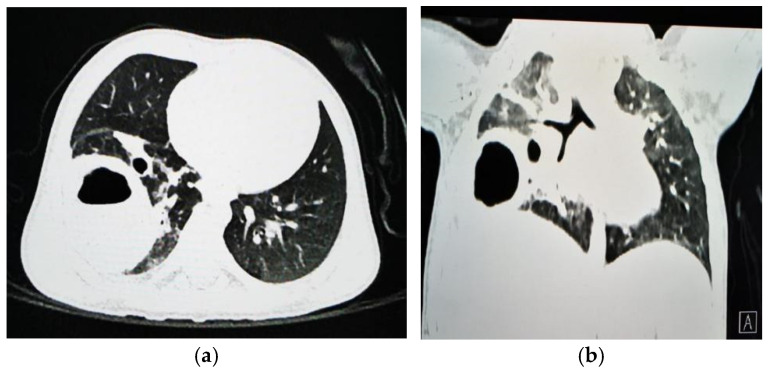
Two-year-old male child with pulmonary abscess: CT scan revealed large mass in right lower lobe, measuring approximately 53 mm × 47 mm × 50 mm, containing heterogeneous contents enclosed by thick walls. Two additional cystic lesions, measuring 13 mm and 7 mm, respectively, observed adjacent to mass without clear demarcation, indicative of multiloculated cystic lesion centered in right lower lobe; (**a**) transverse plane; (**b**) coronal plane.

Despite treatment, fever, pulmonary auscultatory changes, and the inflammatory syndrome, although decreasing, persisted initially (Table 1). A subsequent CT scan performed after 7 days of antibiotic therapy confirmed the presence of the mass in the right lower lobe, measuring approximately 53 mm × 47 mm × 50 mm, with heterogeneous content circumscribed by thick walls. Two additional cystic lesions, measuring 13 mm and 7 mm, respectively, were identified nearby without clear demarcation (Figure 3).

In collaboration with the pediatric surgical team, it was decided to defer surgery and continue intravenous antibiotic therapy, with an ongoing assessment of the patient’s progress. By the tenth day of treatment, the fever had subsided, the cough had improved, and pulmonary auscultation had shown signs of recovery. Inflammatory markers were decreasing, and a thoracic ultrasound revealed a persistent small area of consolidation without evidence of abscess formation. The patient was discharged after completing three weeks of parenteral antibiotic therapy and continued to receive oral antibiotics (Amoxicillin–clavulanate 90 mg/kg/day) at home for an additional two weeks.

**Figure 4:**
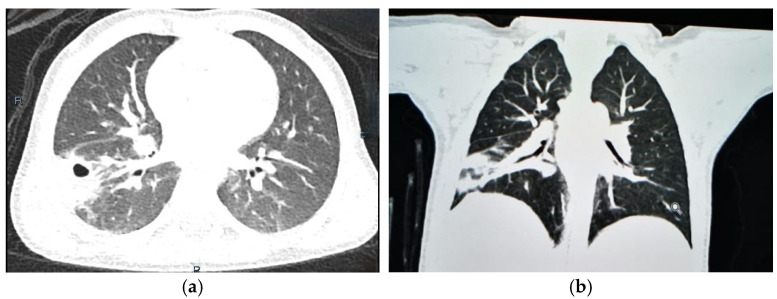
CT scan of 2-year-old male child with pulmonary abscess, demonstrating marked reduction in hydro-aeric cavity in right lower lobe, measuring 22 mm × 22 mm, without associated cystic lesions; (**a**) transverse plane; (**b**) coronal plane.

One month later, the clinical condition was improved with no signs of biological inflammatory syndrome, and a follow-up CT scan revealed a significant reduction in the size of the oval mass in the right lower lobe, as radiological resolution typically occurs at a later stage. There were no associated cystic lesions or signs of congenital pulmonary airway malformation (Figure 4).

**Figure 5:**
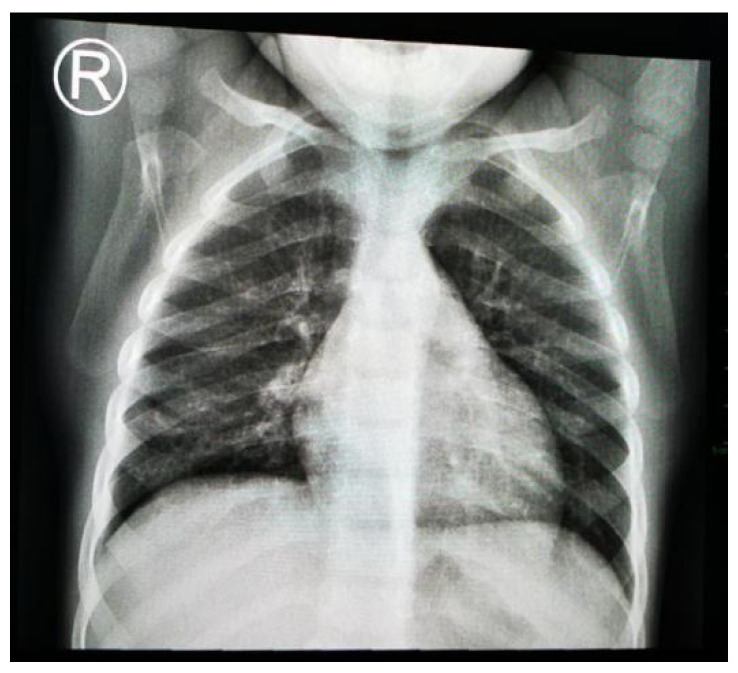
Normal chest X-ray of 2-year-old male child with pulmonary abscess, 3 months post-treatment.

Three months after the CT scan, the pulmonary X-ray was within normal limits (Figure 5), and both clinical and paraclinical evaluations showed no abnormalities. It was concluded that the patient had presented with severe pneumonia that progressed to abscess formation; however, the patient is scheduled to undergo another CT scan in one year for definitive exclusion of the diagnosis of congenital pulmonary airway malformation.

#### 1.9.2. Case 2

A 17-year-old male patient with fever, cough, and right laterothoracic pain was referred to our clinic from a regional hospital. He reported a productive cough that began one month before presentation and gradually worsened, accompanied by dyspnea on exertion and intermittent vomiting. Five days prior to admission, he developed diarrhea and right laterothoracic pain, along with a high fever. He received symptomatic treatment for these symptoms. History of other acute illnesses was not known and he lived in the countryside and took care of domestic animals.

Upon admission, he presented fever and nausea. On respiratory examination, auscultation revealed diminished breath sounds and fine crackles in the right lung, associated with dullness at percussion, with normal oxygen saturation and respiratory rate.

Laboratory tests showed leukocytosis (17,300/µL) with neutrophilia (15,250/µL), and marked indications of inflammatory syndrome (CRP 244 mg/L, procalcitonin 7 ng/mL, ESR 70 mm/h, LDH 630 U/L, fibrinogen 961 mg/dL) with no hypoxemia on arterial blood gas analysis. Liver function tests were slightly elevated (AST 42U/L, ALT 67U/L), but no coagulation disorder was found. Stool and blood cultures, IGRA, and Tuberculin Skin Test were all negative.

**Figure 6:**
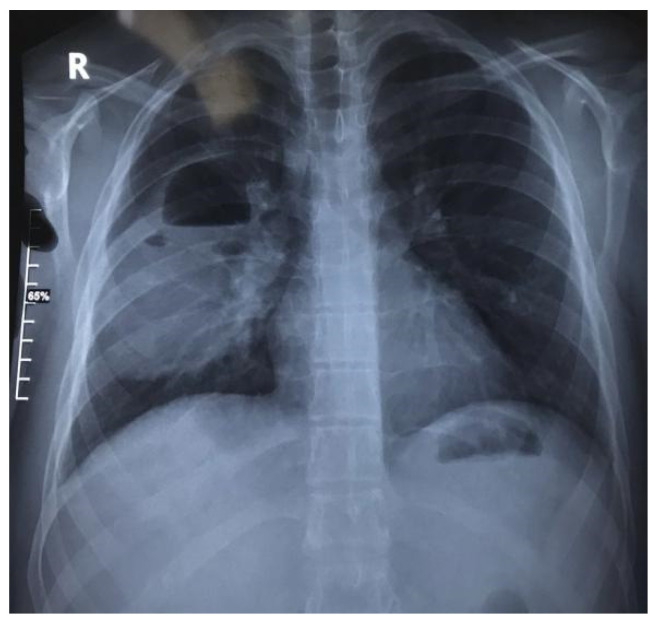
Seventeen-year-old male adolescent with pulmonary abscess: chest X-ray demonstrating oval-shaped, well-circumscribed opacity occupying two-thirds of right hemithorax, characterized by thick walls and containing air–fluid level; satellite lymphadenopathies observed in right hilum.

Chest X-ray revealed an oval-shaped, well-circumscribed opacity in the right hemithorax, with thick walls and containing an air–fluid level. Satellite lymphadenopathies were observed in the right hilum (Figure 6).

After initial clinical, biologicals and imaging evaluations, the diagnoses of necrotising pneumonia with right lung abscess and sepsis were established. The chest X-ray taken upon admission suggested a pulmonary abscess, but a tuberculous cavity, tumor, or an underlying congenital pulmonary airway malformation with secondary infection were also considered. The patient was treated with broad-spectrum antibiotics (Meropenem 3 g/day, Clindamycin 2.25 g/day).

**Figure 7:**
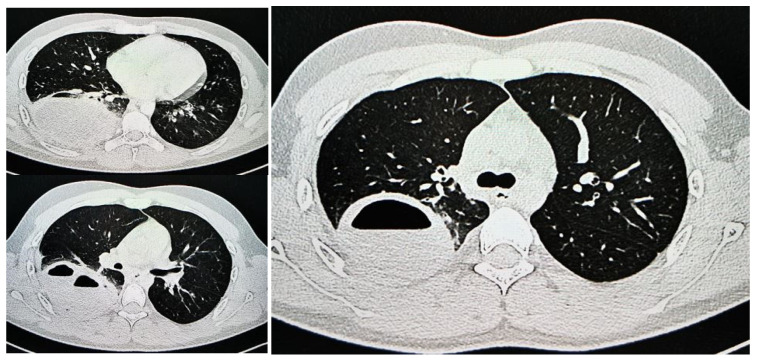
Seventeen-year-old male adolescent with pulmonary abscess: CT scan (transverse plane) with contrast performed, which describes oval mass in inferior right lobe, measuring circa 10 cm × 7 cm × 12 cm, with some round–oval parts containing air–fluid levels, circumscribed by walls and septa with varying thickness; pleural effusion of maximum thickness 10 mm.

The outcome after 7 days of treatment was unfavorable, with persistence of fever and productive cough associated with purulent expectoration, and with marked inflammatory response (Table 2). Due to an unfavorable clinical course, a contrast-enhanced chest CT scan was performed, where an oval mass in the inferior right lobe, measuring circa 10 cm × 7 cm × 12 cm, with some round–oval parts containing air–fluid levels, circumscribed by walls and septa with varying thickness was described, which raised the suspicion of a partially drained hydatid cyst (Figure 7).

Regardless of the negative Echinococcus granulosus antibody test results and the absence of eosinophilia, the decision to initiate Albendazole treatment (800 mg/day) was prompted by clinical suspicion raised by the suggestive radiologic image. Additionally, an abdominal ultrasound was conducted to exclude the presence of a hepatic hydatid cyst.

**Figure 8:**
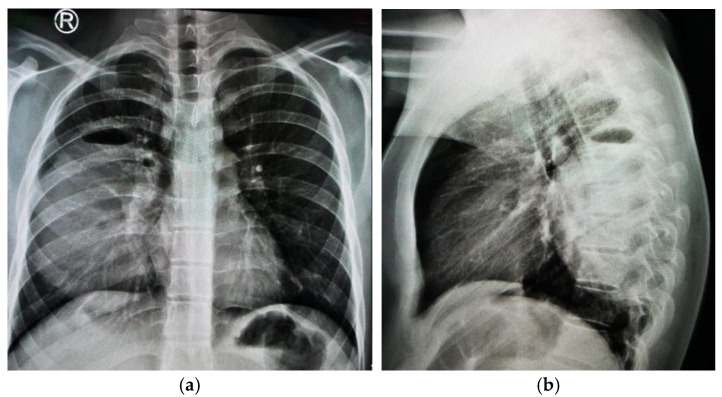
Seventeen-year-old male adolescent with pulmonary abscess: enlargement of oval-shaped mass, measuring approximately 15 cm × 9 cm × 12 cm, characterized by thick walls and containing two air–fluid levels observed in upper third of mass, with increased radiopaque component noted; (**a**) posteroanterior chest X-ray; (**b**) lateral chest X-ray.

Despite administering an additional antiparasitic treatment alongside antibiotic therapy for another week, the patient exhibited poor clinical outcomes, associating hemoptysis, and a persistent inflammatory syndrome (Table 2). A repeated chest X-ray revealed an enlargement of the oval mass in the right lung (Figure 8).

**Figure 9:**
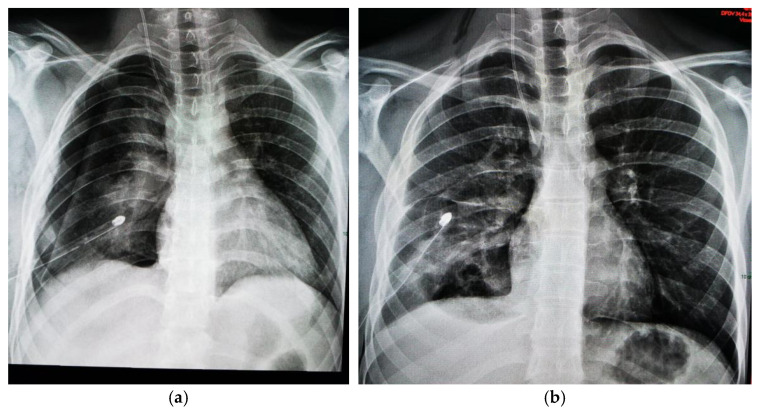
Seventeen-year-old male adolescent with pulmonary abscess: (**a**) post-operative pneumothorax requiring pleural drainage, initially with poor lung expansion; (**b**) after three days, right lung expanded adequately.

Based on these findings, surgical intervention was deemed necessary, leading to a thoracotomy during which the right lower lobe mass was drained. Due to post-operative pneumothorax, pleural drainage was performed, and the initial outcome was slow but favorable, with poor lung expansion necessitating further pleural drainage and aspiration (Figure 9).

**Figure 10:**
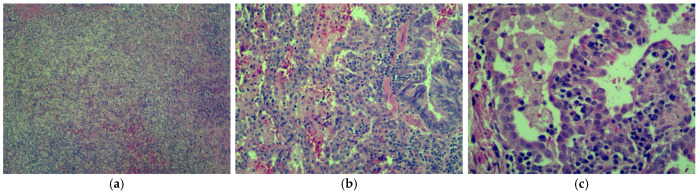
Seventeen-year-old male adolescent with pulmonary abscess—histopathologic results: (**a**) 4× magnification objective: disorganized architecture with alveoli and bronchioles not visible, replaced by fibroblastic reaction and mixed inflammatory infiltrate, indicative of abscess formation; (**b**) 10× magnification objective: mixed inflammatory infiltrate (lymphoplasmacytes, neutrophils, eosinophils, and macrophages) in interalveolar septa and bronchial epithelium; (**c**) 20× magnification objective: alveoli filled with macrophages and thickened interalveolar septa with inflammatory infiltrate consisting of lymphocytes, plasma cells, neutrophils, and eosinophils.

Intraoperatively, the diagnosis of pulmonary cystic echinococcosis was ruled out due to the absence of the proliferative membrane, but a congenital pulmonary airway malformation was suspected, which is why it was sent to pathology for examination (Figure 10). However, histopathologic results did not confirm this suspicion. Due to the fact that a pulmonary abscess results in the destruction of the surrounding parenchyma and making it therefore difficult to determine histopathologically if a pulmonary malformation was present beforehand, a CT scan needs to be repeated after one year to ensure an accurate diagnosis.

During the procedure, green pus was drained from the abscess and samples were collected for culture. However, the cultures were negative.

Postoperatively, a gradual but favorable clinical evolution was observed, with clinical resolution of symptoms and re-expansion of the right lung following pleural drainage. Parenteral antibiotic therapy was continued until discharge for another two weeks, after which the patient received an additional two weeks of orally administered Amoxicillin–clavulanate at 2 g/day.

## 2. Discussions

In our clinical experience, establishing the etiology of pulmonary abscesses in children can be particularly challenging. In both of our cases, repeated blood cultures were negative, failing to identify a specific pathogen. Bronchoalveolar lavage is not commonly performed in our clinic, and sputum cultures are seldom obtained in children because of age-related challenges. Even when sputum samples are collected, distinguishing between colonization and infection is difficult due to the high risk of contamination [10,38]. Despite the lack of microbial identification, the favorable response to conservative treatment in the first case and to surgical drainage in the second case obviated the immediate need for precise etiological determination.

In some cases of pulmonary abscess, bronchoscopy may be necessary to establish the underlying cause and is therefore useful in detecting endobronchial obstruction due to a mass or foreign body, guiding further treatment decisions [3,4].

Neither of the two patients had risk factors for foreign body aspiration [39,40]. In the first case, bronchoscopy was not performed to obtain a tracheobronchial aspirate because the patient responded well to treatment. Although the patient had a history of pneumonia affecting the same lobe, foreign body aspiration was ruled out based on the absence of relevant clinical history and the lack of underlying conditions that would predispose the patient to this diagnosis.

Cultures obtained intraoperatively in the second case were inconclusive, likely due to the previous administration of multiple antibiotic treatments and the inherent difficulty in cultivating bacteria that require specialized media. These challenges underscore the complexity of diagnosing and managing pulmonary abscess in pediatric patients.

In both cases, a CT scan was performed on the seventh day due to the persistence of the inflammatory syndrome and fever, with the aim of determining the subsequent therapeutic approach and identifying any associated complications.

Although abscesses can be associated with pleural effusion or empyema, this was not observed in our patients [1]. In the first case, there was only a minimal pleural reaction, and the patient had a favorable outcome. Chest ultrasound has proven to be very effective in monitoring and diagnosing associated pleural effusions [41].

Our patients required close monitoring, and a follow-up CT scan was recommended 6 to 12 months after the initial imaging to rule out any underlying malformations. This was particularly crucial in the first case, given the history of pneumonia affecting the same lobe.

In our practice, the diagnosis of a pulmonary abscess is typically established through chest X-rays, following clinical and laboratory indications. It is imperative to exclude risk factors or favoring conditions, such as congenital cystic malformations or foreign body aspiration, to ensure accurate diagnosis and appropriate management.

One potential underlying cause is congenital airway malformations like congenital pulmonary airway malformation (CPAM). These may be infected, leading to abscess formation, and are typically determined by imaging studies showing characteristic cystic lesions [42]. Although relatively rare, CPAMs must be considered and excluded, especially in recurrent or persistent lung infections, often necessitating surgical intervention and histopathological examination [22]. 

In the first case, a history of pneumonia affecting the same lobe and characteristic imaging findings, such as two adjacent cysts near the main mass, raised concerns about CPAM. This prompted further evaluation, including a CT scan conducted one month after completing treatment, which ruled out CPAM as the underlying diagnosis. In the second case, the initial suspicion of CPAM arose during intraoperative assessment but the pathological examination did not confirm this suspected diagnosis.

In the second case, pulmonary echinococcosis was considered as a differential diagnosis [43]. The patient, residing in a rural area and managing domestic animals, presented with imaging findings suggestive of a partially evacuated hydatid cyst. Subsequent anti-Echinococcus antibody tests conducted in our laboratory were negative, though these tests are less specific compared to Western blot IgG assays [44]. Despite initiating antiparasitic treatment, the patient’s unfavorable response to the therapy, combined with intraoperative findings, ultimately ruled out pulmonary echinococcosis.

Regarding tuberculosis (TB), its frequent occurrence in our region emphasizes its crucial consideration in the differential diagnosis of pulmonary abscesses. Therefore, in both our cases, IGRA tests and Tuberculin Skin Tests were performed and were negative. Tuberculosis should be particularly considered in regions with high prevalence or in children with TB exposure [45]. Symptoms such as chronic cough, weight loss, fever, and night sweats are nonspecific and may not always be present, while imaging may reveal cavitary lesions resembling abscesses [46].

## 3. Conclusions

Pulmonary abscesses are rarely encountered in pediatric patients but they can appear secondarily to predisposing factors such as foreign body aspiration, cystic pulmonary malformations, pulmonary infections (e.g., tuberculosis), malignancies, and immune deficiencies. The successful management of pulmonary abscesses relies heavily on early diagnosis and a collaborative approach involving a multidisciplinary team. With prompt and appropriate treatment, the prognosis is generally positive, provided there are no underlying predisposing conditions.

## Figures and Tables

**Table 1:** Case study 1: Two-year-old male child with pulmonary abscess. Laboratory results.

	On Admission	7th Day of Treatment	10th Day of Treatment	Normal Range
Complete blood count				
Leucocytes	19,600/μL	17,950/μL	15,400/μL	6–17 × 10^3^/μL
Lymphocytes	4400/μL	3100/μL	4300/μL	3–9.5 × 10^3^/μL
Monocytes	1160/μL	1050/μL	1180/μL	0–1 × 10^3^/μL
Neutrophils	9900/μL	8800/μL	8600/μL	1.5–8.5 × 10^3^/μL
Platelets	621,000/μL	520,000/μL	560,000/μL	150,000–450,000/μL
Hemoglobin	9.5 g/dL	10.2g/dL	10.8 g/dL	11–14 g/dL
MCV	70/fL	72.1/fL	73/fL	73–89/fL
MCHC	29.6 g/dL	31 g/dL	32.9 g/dL	32–37 g/dL
Inflammatory markers				
CRP	120.4 mg/L	80 mg/L	63 mg/L	0–5 mg/L
Procalcitonin	5.9 ng/mL	0.08 ng/mL		<0.05 ng/mL
LDH	238 U/L			120–300 U/L
Coagulation				
PT	15.9 s			11.3–15.6 s
APTT	32.5 s			24–37 s
INR	1.19			0.84–1.2
Liver function				
AST	29.4 U/L			2–59 U/L
ALT	16.2 U/L			2–29 U/L
Kidney function				
Creatinine	0.19 mg/dL			<0.47 mg/dL
BUN	9.6 mg/dL			<41 mg/dL
Serum iron	10.7 μg/dL			29–91 μg/dL
Serum protein	7.06 g/dL			5.6–7.5 g/dL

MCV: mean corpuscular volume; MCHC: mean corpuscular hemoglobin concentration; CRP: C-reactive protein; LDH: lactate dehydrogenase; PT: prothrombin time; APTT: activated partial thromboplastin time; INR: international normalized ratio; AST: aspartate aminotransferase; ALT: alanine transaminase; BUN: blood urea nitrogen.

**Table 2:** Case study 2: Seventeen-year-old male patient with pulmonary abscess. Laboratory results.

	On Admission	7th Day of Treatment	15th Day of Treatment	Normal Range
Complete blood count				
Leucocytes	17,300/μL	17,900/μL	19,900/μL	4.5–13 × 10^3^/μL
Lymphocytes	740/μL	1100/μL	1900/μL	1.5–6.5 × 10^3^/μL
Monocytes	1250/μL	900/μL	2000/μL	0–1 × 10^3^/μL
Neutrophils	15,250/μL	16,500/μL	15,500/μL	1.8–8.0 × 10^3^/μL
Eosinophils	600/μL	500/μL	300/μL	0–0.7 × 10^3^/μL
Platelets	352,000/μL	420.000/μL	350,000/μL	150,000–450,000/μL
Hemoglobin	14 g/dL	13.6 g/dL	13.7 g/dL	11.7–16.6 g/dL
Inflammatory markers				
ESR	70 mm/1 h			2–15 mm/1 h
CRP	244 mg/L	201 mg/L	191 mg/L	0–5 mg/L
Procalcitonin	7 ng/mL		1.5 ng/mL	<0.05 ng/mL
LDH	630 U/L			120–300 U/L
Fibrinogen	961 mg/dL			160–390 mg/dL
Coagulation				
PT	16.2 s		16.3 s	11.3–15.6 s
APTT	27.5 s		28 s	24–37 s
INR	1.24		1.25	0.84–1.2
Liver function				
AST	42 U/L		19 U/L	2–40 U/L
ALT	67 U/L		27 U/L	2–41 U/L
Kidney function				
Creatinine	0.72 mg/dL		0.7 mg/dL	<1.2 mg/dL
BUN	25 mg/dL		43 mg/dL	<41 mg/dL

ESR: erythrocyte sedimentation rate; CRP: C-reactive protein; LDH: lactate dehydrogenase; PT: prothrombin time; APTT: activated partial thromboplastin time; INR: international normalized ratio; AST: aspartate aminotransferase; ALT: alanine transaminase; BUN: blood urea nitrogen.

## Data Availability

Data is contained within the article.
